# Multimorbidity, Disadvantage, and Trends in Disability-Free Life Expectancy: The CFAS Studies

**DOI:** 10.1093/geroni/igaa057.2200

**Published:** 2020-12-16

**Authors:** Andrew Kingston, Holly Bennett, Louise Robinson, Lynne Corner, Carol Brayne, Fiona Matthews, Carol Jagger

**Affiliations:** 1 Newcastle University, Newcastle upon Tyne, England, United Kingdom; 2 Cambridge Institute of Public Health, University of Cambridge, Cambridge, England, United Kingdom

## Abstract

The combined contribution of multi-morbidity and socio-economic position (SEP) to trends in disability free life expectancy (DFLE) is unknown. We use longitudinal data from the Cognitive Function and Ageing Studies (CFAS I: 1991; CFAS II: 2011), with two year follow up. Disability was defined as difficulty in activities of daily living, and SEP as area-level deprivation. Multi-morbidity was constructed from nine self-reported health conditions and categorised as 0-1, 2-3, 4+ diseases. In 1991 and 2011, shorter total and disability-free years were associated with greater multi-morbidity. Between 1991 and 2011, gains in life expectancy and DFLE were observed at all levels of multi-morbidity, the greatest gain in DFLE being 4 years for men with 0-1 diseases. As multi-morbidity is more prevalent in more disadvantaged groups, further analyses will investigate whether SEP differences remain at all levels of multi-morbidity.

